# Suppression of 4.1R enhances the potency of NKG2D-CAR T cells against pancreatic carcinoma via activating ERK signaling pathway

**DOI:** 10.1038/s41389-021-00353-8

**Published:** 2021-09-21

**Authors:** Yaoxin Gao, Haizhen Lin, Dandan Guo, Sijia Cheng, Ying Zhou, Li Zhang, Jie Yao, Muhammad Asad Farooq, Iqra Ajmal, Yixin Duan, Cong He, Lei Tao, Shijia Wu, Mingyao Liu, Wenzheng Jiang

**Affiliations:** Shanghai Key Laboratory of Regulatory Biology, School of Life Sciences, East China Normal University, 200241 Shanghai, China

**Keywords:** Cancer prevention, Immunotherapy

## Abstract

Pancreatic carcinoma (PC) is one of the most common malignancies. Chimeric antigen receptor (CAR)-modified T cells has achieved remarkable efficacy in the treatment of hematological malignancies. However, lack of tumor-specific targets and the existence of inhibitory factors limit the function of CAR T cells when treating solid tumors. 4.1R has been reported to suppress the anti-tumor activity of T cell responses. In this study, we investigated the anti-tumor activity of 4.1R deletion in natural killer group 2D (NKG2D)-CAR T cells against PC. The CAR T cells were obtained by transfecting T cells with lentiviral vector carrying NKG2D-CAR, NC-NKG2D-CAR, or KD2-NKG2D-CAR. In vitro, NKG2D-CAR T cells showed higher cytotoxicity than Mock T cells. However, compared to NKG2D-CAR T cells, furtherly higher cytotoxicity against PC cells in a dose-dependent manner was found in KD2-NKG2D-CAR T cells. In addition, the proliferation rate and cytotoxic activity of KD2-NKG2D-CAR T cells were significantly higher than those of NKG2D-CAR T cells. Besides, the inhibitory receptors PD-1 and TIM-3 were expressed in lower level on KD2-NKG2D-CAR T cells. In vivo, KD2-NKG2D-CAR T cells suppressed tumor growth more effectively in a xenograft model compared to NKG2D-CAR T cells. Mechanistically, 4.1R regulated CAR T cell function via activating ERK signaling pathway. Therefore, the study provides a new idea to enhance the anti-tumor efficiency of CAR T therapy.

## Introduction

Pancreatic carcinoma (PC) is one of the most metastatic and deadly cancers, with a 5-year survival rate of less than 5% [1, 2]. So far, radical surgery is one of the best options to cure PC, but it is difficult to diagnose when surgical resection is feasible [3]. Therefore, most patients will finally develop local and/or metastatic recurrence, which results in high mortality and incidence as well as poor prognosis [4].

With the rapid development of cancer immunotherapy, adoptive transfer of chimeric antigen receptor T cells (CAR T) can relieve hematologic malignancies in the long term [5]. Currently, the treatment of B-cell malignancies using CD19 CAR T cell therapies has been approved by FDA [6]. However, there are still a lot of challenges in the therapeutic of solid tumors due to the scarcity of tumor-specific targets.

Natural killer group 2D (NKG2D), an activating receptor expressed on many immune effector cells, has an important role in tumor surveillance. There are eight types of NKG2D ligands (NKG2DLs) including MHC I chain-related molecules A and B (MICA and MICB) and six cytomegalovirus UL16-binding proteins (ULBP1–6). It is well-established that NKG2DLs are primarily expressed on most types of tumor cells, including hematological and solid tumors, but are normally absent or expressed in low levels on healthy tissues [7, 8]. The characters indicate the NKG2D receptor can be used as a potential target in immunotherapy for treating tumors. Up to now, NKG2D CAR T cells have been applied in both hematologic and solid tumor treatment in clinical practice. The safety and feasibility of NKG2D-CAR T cells were evaluated and the researchers found out that the proliferation and persistence of NKG2D-CAR T cells were restricted in vivo. Therefore, further modifications are needed to boost clinical effect although NKG2D-CAR T cells presented marvelous outcomes to lysis tumor cells in vitro [9–11].

Cytoskeletal protein 4.1R (4.1R), belonging to 4.1 family, includes three highly conserved domains: an N-terminal membrane-binding domain (MBD), an internal spectrin-actin-binding domain (SABD), and a C-terminal domain (CTD) [12]. It is firstly identified in red cells where it plays an important role in maintaining the mechanical stability of red cell membranes [13, 14]. Currently, studies have reported that the 4.1R exerts its effect by binding directly to LAT, and thereby inhibiting phosphorylation of ZAP-70 in T cells [15].

In this study, we engineered the CAR T with NKG2D and shRNA-4.1R to treat PC. Our data showed that cell proliferation, cytotoxicity, and granzyme expression were significantly increased and the exhaustion markers were observably decreased in KD2-NKG2D-CAR T cells compared to NKG2D-CAR T cells in vitro. Consistently, KD2-NKG2D-CAR T cells indicated stronger anti-antitumor activity in vivo. These findings provide a new tool to enhance the efficiency of CAR T cell therapy.

## Results

### Targeting 4.1R with shRNA leads to efficient 4.1R knockdown in NKG2D-CAR T cell

**Fig. 1:**
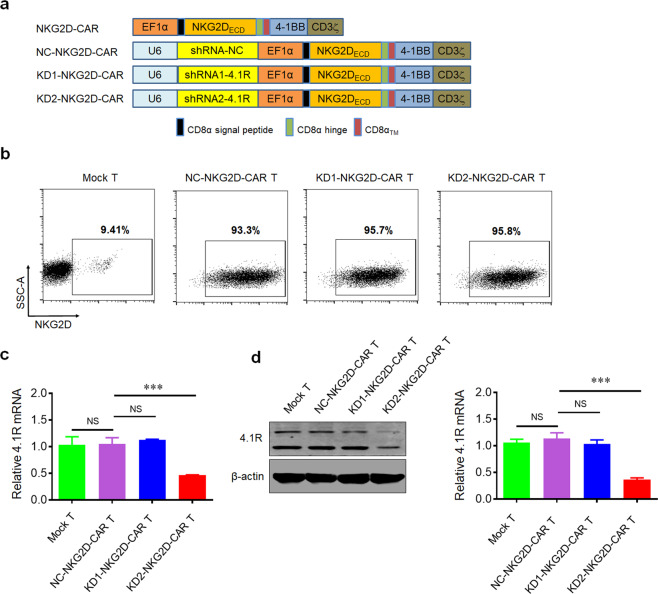
Generation and characterization of NKG2D-CAR T cells with 4.1R knockdown. **a** Schematic representation of NKG2D-targeted constructs with CD28 and 4-1BB costimulatory domain, a CD3ζ domain, and a shRNA-4.1R. **b** Flow cytometry analysis of the transduction efficiencies. T cells were transfected by the lentiviruses expressing NKG2D-CAR and the transfection efficiency was analyzed by FACS after the cells were stained with fluorescently-labeled anti-NKG2D antibodies. **c** Quantitative real-time PCR analysis of 4.1R mRNA level in Mock T cells, NC-NKG2D-CAR T cells, KD1-NKG2D-CAR T cells, and KD2-NKG2D-CAR T cells. Total mRNAs of the transfected cells were extracted and reversely transcribed, the expression level of 4.1R was analyzed by quantitative real-time PCR. **d** Western blot analysis of 4.1R protein level in Mock T cells, NC-NKG2D-CAR T cells, KD1-NKG2D-CAR T cells, and KD2-NKG2D-CAR T cells. Data were representative of three independent experiments. ***P < 0.001, NS not significant.

The second-generation CAR backbone was used to construct CAR T cells. The CAR construct contained CD8α leader, extracellular domain of NKG2D, hinge, and transmembrane domain (TM) of CD8α, cytoplasmic domain of 4-1BB (CD137), cytoplasmic tail of CD3ζ, and the shRNA targeting protein 4.1R (shRNA-4.1R), and the construct was named as KD1-NKG2D CAR or KD2-NKG2D CAR (Fig. 1a). The Mock T cells were generated by transducing an empty lentivirus, and NKG2D-CAR T cells was prepared by transducing lentiviruses expressing NKG2D-CAR and NC-NKG2D-CAR or KD-NKG2D-CAR. Anti-NKG2D antibody was used to measure the expression level of NKG2D in CAR T cells by flow cytometry using BD LSRFortessa, and the result showed that the expression level of NKG2D in CAR T cells was significantly higher than Mock T cells, indicating that NKG2D-based CAR was successfully transduced in T cells (Fig. 1b).

To knock down the expression of protein 4.1R in NKG2D-CAR T cells, shRNA-4.1R was co-expressed. The expression of 4.1R at transcriptional and protein level was analyzed by quantitative real-time PCR and Western blot. The results showed that the expression of 4.1R in KD2-NKG2D-CAR T cells was significantly lower than that in NC-NKG2D-CAR T cells, while there was no statistical difference between KD1-NKG2D-CAR T cells and NC-NKG2D-CAR T cells or Mock T cells (Fig. 1c, d). Thus, KD2-NKG2D-CAR T cells were used in the following studies.

### Knockdown of 4.1R enhances the cytotoxic ability and the activation of NKG2D-CAR T cells in vitro

**Fig. 2:**
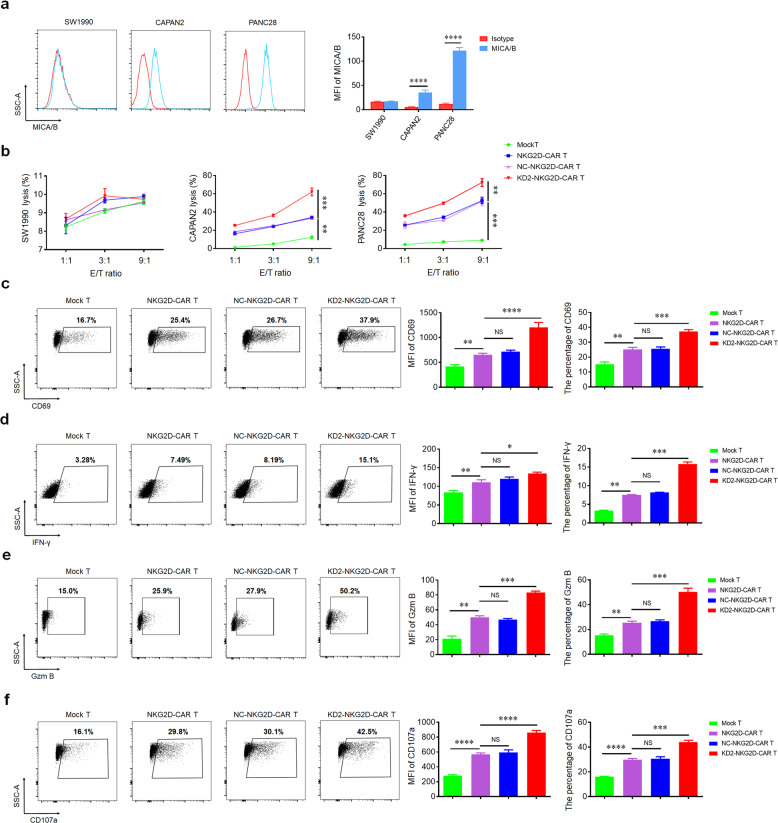
The expression of NKG2DLs in pancreatic carcinoma cell lines and cytotoxic activity of NKG2D-CAR T cells. **a** Flow cytometry histograms showed the expression of NKG2DLs on SW1990, CAPAN2, and PANC28. **b** Line plots displayed the cytotoxicity of Mock T, NKG2D-CAR T, NC-NKG2D-CAR T, and KD2-NKG2D-CAR T against the indicated cell lines at a different effector to target (*E*:*T*) ratios for 16 h. The expression of CD69 (**c**), IFN-γ (**d**) and Gzm B (**e**) as well as the expression of CD107a (**f**) were measured by flow cytometry after co-incubating with PANC28 at a 9:1 ratio for 16 h (left). MFI and percentage were statistically analyzed and shown in column chart (middle and right) (*n* = 3). Data were representative of three independent experiments. *P < 0.05, **P < 0.01, ***P < 0.001, ****P < 0.0001, NS not significant.

The expression level of NKG2DLs on the cancer cell surface is the key factor influencing the antitumor activity of NKG2D-CAR T cells. Hence, flow cytometry was used to detect the distribution of MICA/B on SW1990, CAPAN2, and PANC28, respectively. Our data showed that many PC cell lines expressed high levels of NKG2DLs. In these cell lines, the highest expression was detected on the surface of PANC28, while the lowest expression was observed on the surface of SW1990, which was used as a negative control in the subsequent experiments (Fig. 2a). To evaluate the anti-tumor activity of NKG2D-CAR T cells in vitro, we co-incubated NKG2D-CAR T cells and different PC cell lines at the different ratio of 1:1, 3:1, and 9:1. All types of NKG2D-CAR T cells exerted significantly cytotoxic activity against CAPAN2 and PANC28 but not SW1990. As expected, KD2-NKG2D-CAR T group revealed higher cytotoxic activity compared to NKG2D-CAR T group and NC-NKG2D-CAR T group. There was no significant difference between NKG2D-CAR T group and NC-NKG2D-CAR T group (Fig. 2b).

### Knockdown of 4.1R spurs the activation, degranulation, and cytokine secretion of NKG2D-CAR T cells

CD69, a T cell surface molecule, is regarded as the activation marker [16]. After the coculture of NKG2D-CAR T cells with PANC28 and CAPAN2, the expression of CD69 in NKG2D-CAR T cells was dramatically increased compared to Mock T cells (Fig. 2c and Supplementary Fig. 1a), but not SW1990 (Supplementary Fig. 2a). Interestingly, higher expression of CD69 was observed in KD2-NKG2D-CAR T group compared to NKG2D-CAR T group and NC-NKG2D-CAR T group. There was no significant difference between NKG2D-CAR T group and NC-NKG2D-CAR T group.

As T cell-mediated cytotoxicity was largely dependent on IFN-γ and Gzm B [17]. We assessed the expression of IFN-γ and Gzm B. Consistent with CD69, the expression of IFN-γ and Gzm B in NKG2D-CAR T cells were notably increased compared to Mock T cells after co-culturing with PANC28 and CAPAN2. Higher level of IFN-γ and Gzm B was observed in KD2-NKG2D-CAR T group compared to NKG2D-CAR T group and NC-NKG2D-CAR T group (Fig. 2d, e and Supplementary Fig. 1b, c), but not SW1990 (Supplementary Fig. 2b, c). There was no significant difference between NKG2D-CAR T group and NC-NKG2D-CAR T group.

Degranulation, an indicator of the lytic function of T lymphocytes, can be quantified by detection of CD107a [18]. A higher level of CD107a was detected in NKG2D-CAR T cells compared to Mock T cells after co-culturing with PANC28 and CAPAN2. Besides, the expression of CD107a in KD2-NKG2D-CAR T group was higher than NKG2D-CAR T group and NC-NKG2D-CAR T group (Fig. 2f and Supplementary Fig. 1d), but not SW1990 (Supplementary Fig. 2d). There was no significant difference between NKG2D-CAR T group and NC-NKG2D-CAR T group.

### Knockdown of 4.1R promotes the cell proliferation and reduces the exhaustion of NKG2D-CAR T cells

**Fig. 3:**
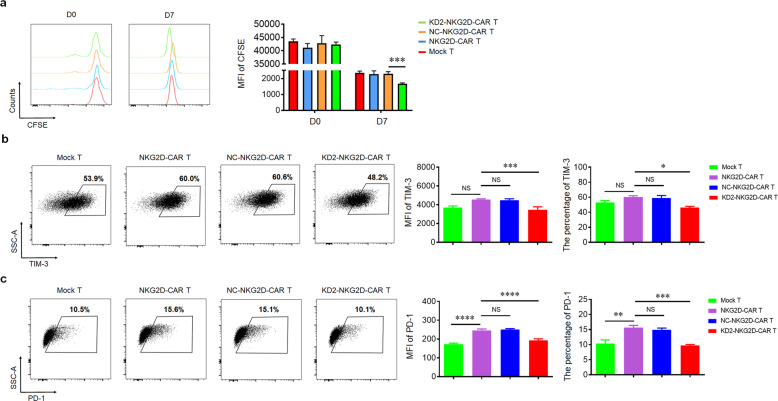
4.1R deficiency enhanced cell proliferation and reduced exhaustion of CAR T cells. **a** Mock T, NKG2D-CAR T, NC-NKG2D-CAR T, and KD2-NKG2D-CAR T, labeled with CFSE, were co-incubated with PANC28 at a 1:5 ratio for 7 days. CFSE dilution was used as a measure of cell proliferation (left), and MFI was calculated (right) (*n* = 3). **b**, **c** Mock T, NKG2D-CAR T, NC-NKG2D-CAR T, and KD2-NKG2D-CAR T were co-incubated with PANC28 at a 1:5 ratio for 3 days. Anti-CD3 staining was used to distinguish T cells from target cells. The expression of TIM-3 and PD-1 was measured by flow cytometry (left). MFI and percentage were statistically analyzed and shown in column chart (middle and right) (*n* = 3). Data were representative of three independent experiments. *P < 0.05, **P < 0.01, ***P < 0.001, ****P < 0.0001, NS not significant.

To investigate the functionality of NKG2D-CAR T cells and KD2-NKG2D-CAR T cells, this study examined the proliferative capability after co-culturing with PANC28. KD2-NKG2D-CAR T group proliferated much more rapidly than other groups; and there was no significant difference among Mock T group, NKG2D-CAR T group, and NC-NKG2D-CAR T group (Fig. 3a).

Many inhibitory receptors including PD-1 and TIM-3 expressing on T lymphocytes could lead to decreased ability to kill and secrete cytokines [19, 20]. After co-culturing with PANC28, the expression of PD-1 in NKG2D-CAR T cells was dramatically increased compared to Mock T cells, but not TIM-3. Interestingly, lower expression of both PD-1 and TIM-3 was observed in KD2-NKG2D-CAR T group compared to NKG2D-CAR T group and NC-NKG2D-CAR T group. There was no significant difference between NKG2D-CAR T group and NC-NKG2D-CAR T group (Fig. 3b, c).

### 4.1R regulates NKG2D-CAR T cells via ERK signaling pathway

**Fig. 4:**
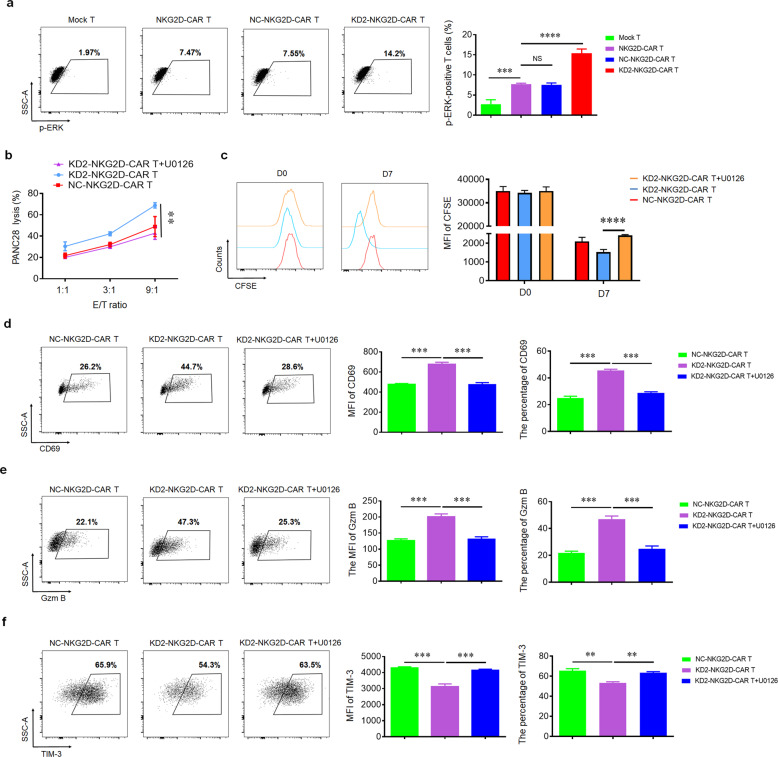
4.1R deficiency regulated the function of CAR T cells via ERK signaling pathway. **a** Mock T, NKG2D-CAR T, NC-NKG2D-CAR T, and KD2-NKG2D-CAR T were co-incubated with PANC28 at a 9:1 ratio for 16 h. The expression of p-ERK was detected by flow cytometry (left), and the percentage of p-ERK-positive T cells was statistically analyzed (right) (*n* = 3). **b** Line plots displayed the cytotoxicity of NC-NKG2D-CAR T and KD2-NKG2D-CAR T against PANC28 at a different effector to target (*E*:*T*) ratios for 16 h in the absence and presence of 10 μM U0126. **c** NC-NKG2D-CAR T and KD2-NKG2D-CAR T were co-incubated with PANC28 at a different effector to target (*E*:*T*) ratios for 7 days in the absence and presence of 10 μM U0126. CFSE dilution was used as a measure of cell proliferation (left), and MFI was calculated (right) (*n* = 3). NC-NKG2D-CAR T and KD2-NKG2D-CAR T were co-incubated with PANC28 at a different effector to target (*E*:*T*) ratios for 16 h in the absence and presence of 10 μM U0126. The expression of CD69 (**d**), Gzm B (**e**), and TIM-3 (**f**) was detected by flow cytometry (left). MFI and percentage were statistically analyzed and shown in column chart (middle and right) (*n* = 3). Data were representative of three independent experiments. **P < 0.01, ***P < 0.001, NS not significant.

ERK, a member of MAPK pathways, has been reported playing an important role in 4.1R^−/−^ T cells [15]. In this study, we found that after downregulating 4.1R, the expression of p-ERK was upregulated (Fig. 4a). To elucidate whether 4.1R regulated NKG2D-CAR T cell function by activating ERK, p-ERK inhibitor U0126 was added, and the data showed that the expression of p-ERK was inhibited (Supplementary Fig. 3), resulting in the suppression of the cytotoxic function, cell proliferation, and CD69 expression (Fig. 4b–d). In addition, the production of IFN-γ (data not shown) as well as Gzm B production (Fig. 4e), and CD107a expression (data not shown) were inhibited after blocking the ERK signaling pathway. On the contrary, the expression of TIM-3 (Fig. 4f) and PD-1 (data not shown) was upregulated in response to the inhibitor. The results indicated that 4.1R mediated the function of NKG2D-CAR T cells via ERK signaling pathway.

### 4.1R-silencing NKG2D-CAR T cells show more effective anti-tumor activity in mice

**Fig. 5:**
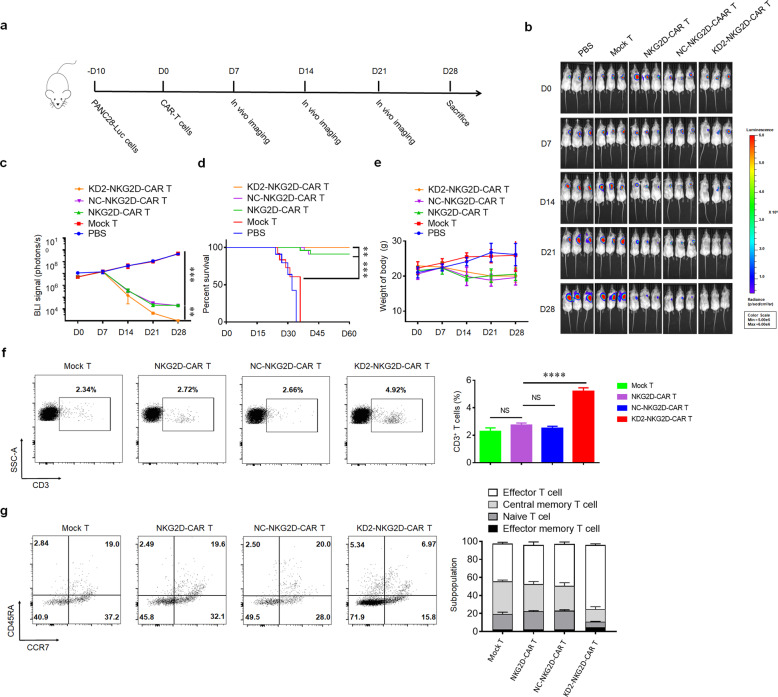
4.1R deficiency showed effective and persistent anti-tumor activity against xenografts formed by PANC28 in mice. **a** Schematic diagram of a complete animal experiment. **b**, **c** Tumor bioluminescence images of mice transplanted with PANC28/luc cells at the indicated time points. **d** Overall survival of mice was presented in Kaplan–Meier curves. **e** Line plots displayed the body weight of mice. **f** The counts of CD3^+^ T cells in peripheral circulation were measured by flow cytometry (left), and the percentage of CD3^+^ T cells was calculated (right) (*n* = 3). **g** The ratio of the subpopulation of NKG2D-CAR T cells in peripheral circulation was measured by flow cytometry. Data were representative of three independent experiments. **P < 0.01, ***P < 0.001, ****P < 0.0001, NS not significant.

To evaluate the therapeutic efficacy of NKG2D-CAR T cells in vivo, subcutaneous xenografts were established by injecting stable luciferase-transfected PANC28 cells in NSG mice. Around 10 days later, the mice were randomly divided into five groups and then subjected to intravenous injections of phosphate-buffered saline (PBS), Mock-T cells, NKG2D-CAR T cells, NC-NKG2D-CAR T cells, and KD2-NKG2D-CAR T cells (Fig. 5a). Bioluminescence imaging showed that sustained tumor regression was observed for NKG2D-CAR T group compared with the PBS and Mock T groups. In addition, KD2-NKG2D-CAR T group showed a significantly reduced tumor volume and better survival compared with NKG2D-CAR T and NC-NKG2D-CAR T groups (Fig. 5b–d). The lower body weight of the mice was measured in NKG2D-CAR T groups compared to PBS and Mock T groups, while no significance difference was observed among NKG2D-CAR T groups (Fig. 5e). To quantify the persistence of infused human T cells, peripheral blood was collected from tumor-bearing mice. Twenty-eight days after the first injection of CAR T cells, the human CD3^+^ T cell counts in KD2-NKG2D-CAR T group and the ratio of effector T cell were higher than other groups (Fig. 5f, g). These data suggested 4.1R deletion enhanced the anti-tumor function of NKG2D-CAR T cells in vivo.

## Discussion

Pancreatic carcinoma (PC) has become one of the most lethal cancers in the world and the clinical treatment for PC is mainly surgical resection. However, a lot of PC patients are often diagnosed at an advanced stage, which makes it very difficult to be treated at the appropriate time [2, 3]. In current years, the emergence of CAR T immunotherapy provides a promising approach for the therapy of malignant tumors. Although a better treatment effect of CAR T has been found in B-cell malignancies, a number of factors restrict the treatment of CAR T for solid tumors [6, 7].

“Off-tumor, on target” is one of the most notable factors for CAR T cell to treat solid tumor [21]. NKG2D is an activating receptor expressed on T and NK cells and the expression of NKG2DLs on PC and other tumors, and immunosuppressive cells have been confirmed [22]. Hence, NKG2D-CAR T cells targeting NKG2DLs have been designed to eliminate various tumors [23]. Based on these results, we designed second-generation NKG2D-CAR T cells to treat PC.

To further improve the antitumor activity of CAR T cells, a number of strategies to increase the efficacy of CAR T cells have been applied, such as enhancing chemokine receptor expression, or adding antibody to block checkpoint receptor. However, these strategies do not seem to have much effect against PC tumors [24, 25]. Hence, some inhibitory genes have been knocked down in CAR T cells to enhance the function of CAR T cells, such as A2aR, ACAT1, and PD-1 [26–28]. Cytoskeletal protein 4.1R, one member of 4.1 family, has been reported to have the function of inhibiting the activity of T cells [15]. In this study, the effects of 4.1R gene knockdown were investigated in NKG2D-CAR T cells. The NKG2D-CAR T cells containing shRNAs were generated to interfere with the expression of 4.1R. As expected, the reduction of 4.1R resulted in enhanced cytotoxic functions of NKG2D-CAR T cells. Besides, IFN-γ and Gzm B, mediating the cytotoxicity of T cells, were upregulated obviously in KD2-NKG2D-CAR T cells compared to NKG2D-CAR T cells and control T cells. The expression of CD69 and CD107a, sensitive activation indicators for T cell function, was higher in KD2-NKG2D-CAR T group compared to NKG2D-CAR T cells and control T cells. T-cell accumulation in tumor contributes to suppressing the tumor growth [29]. Here, we validated the effect of 4.1R inhibition on cell proliferation, and we found that KD2-NKG2D-CAR T cells could proliferate much more rapidly than NKG2D-CAR T cells and control T cells. High expression of TIM-3 and PD-1 on T cells, indicating that T cells are in depleted state, impairs the function of T cells against tumor [19, 20]. In our study, our data showed that lower expression of TIM-3 and PD-1 was detected in KD2-NKG2D-CAR T cells compared to NKG2D-CAR T cells and control T cells. Moreover, the stronger antitumor effect of NKG2D-CAR T cells with silencing of 4.1R was found in PC xenograft tumor models. Our results validated that inhibiting 4.1R could enhance the function of NKG2D-CAR T cells, providing a potential therapeutic strategy for the clinical treatment of PC. In addition, our finding demonstrated that 4.1R regulated the function of NKG2D-CAR T cells through ERK signaling pathway and shed new light on the mechanisms of 4.1R-silencing NKG2D-CAR T cells.

Both RNA interference (RNAi) technology and CRISPR/cas9 technology are extensively used to suppress the gene expression [30, 31]. RNAi is widely used to knock down the expression of a target gene through transient expression of a small interfering RNA (siRNA), or through stable expression of a short hairpin RNA (shRNA) [32]. CRISPR/cas9 technology has emerged as a powerful approach to knock out the target gene by Cas9-mediated DNA cleavage in recent years [33]. However, shRNA is more convenient and affordable compared with CRISPR/cas9. In current study, shRNA and CAR were constructed in one vector and the CAR T cells with 4.1R gene knockdown were successfully acquired by infection of the lentiviruses co-expressing shRNA and CAR.

Altogether, this study indicated that knockdown of 4.1R could activate NKG2D-CAR T cells and increase the release of INF-γ and Gzm B via ERK signaling pathway, thereby, enhancing the anti-tumor activity of NKG2D-CAR T cells against pancreatic carcinoma. Besides, our findings provided the potential application of modified CAR T cells for treatment of solid malignancies in clinic.

## Materials and methods

### Cell cultures

SW1990, CAPAN2, and PANC28 cells were purchased from American Tissue Culture Collection (ATCC, USA) and cultured in Modified Eagle Medium (DMEM) (Gibco Laboratories, Grand Island, NY) supplemented with 10% fetal bovine serum (FBS) (Gibco Laboratories) and 1% penicillin-streptomycin, which were incubated in a humidified incubator with 5% CO_2_ at 37 °C for several days. The medium was changed every 2 days.

### shRNA design

Human 4.1R mRNA (Accession: NM_001166005) was used as the template strand, and the target gene interference sequence was obtained using an online shRNA design tool (http://rnaidesigner.thermofisher.com/rnaiexpress/sort.do). In our study, we designed two 4.1R target sequences and a negative sequence to construct the lentiviral shRNAs. The sequences of shRNAs are shown in Table 1. shRNA fragments were synthesized by Invitrogen (Shanghai, China), and an EcoR І cleavage site was inserted at the end of shRNA. Two complementary nucleic acid strands targeting interference sequence were synthesized and cloned into the target vector. Then, they were confirmed using specific enzyme digestion and agarose gel electrophoresis.

**Table 1:** shRNA sequences for targeting 4.1R transcripts.

Name	Sense sequence	Anti-sense sequence	Target gene
shRNA-NC	GAACCCACCTCCAGTAAATGG	CCATTTACTGGAGGTGGGTTC	4.1R-NM_001166005
shRNA-1 (KD1)	GACAGTACCCACCTCAAATGG	CCATTTGAGGTGGGTACTGTC	
shRNA-2 (KD2)	GTGACAGTACCCACCTCAAAT	ATTTGAGGTGGGTACTGTCAC	

### CAR preparation

To target NKG2DLs, we synthesized extracellular domain of human NKG2D (Idobio, China), and created the 2nd generation CAR T cells, human NKG2D-CAR, consisting of the CD8α signal peptide, NKG2D, CD8α hinge and TM, 4-1BB (CD137) cytoplasmic domain, CD3ζ cytoplasmic tail, and the sequence of shRNA targeting protein 4.1R and negative control (NC) were subcloned into upstream of NKG2D-CAR. Lentiviral particles were generated by co-transfecting lentiviral plasmids and the packaging plasmids (psPAX2 and pMD2.G) into HEK293T cells at a ratio of 5:5:3. The transfected cells were incubated at 37 °C for 6–8 h, and then in fresh medium for another 48 h. Supernatants were collected, filtered through a 45 μm filter, and concentrated by ultracentrifugation at 15,000 rpm for 2.5 h at 4 °C. The viruses were aliquoted and stored at −80 °C.

### CAR T cell preparation

T cells were isolated from peripheral blood mononuclear cells (PBMCs) by Ficoll-Paque PLUS gradient centrifugation (GE Healthcare) from peripheral blood of healthy volunteer donors (Tianjin Haoyang Biological Manufacture Co., Ltd., China). Primary T cells were sorted using a Pan T Cell Isolation Kit (Miltenyi Biotech) through negative selection from PBMCs according to the manufacturer’s instructions. T cells were cultured in X-VIVO 15 medium containing 5% FBS and 200 U/mL IL-2 and stimulated by magnetic beads coated with agonist antibodies against CD3 and CD28 for 48 h. After 48 h of expansion, T cells were transduced with NKG2D-CAR by adding lentiviral particles and polybrene (8 µg/mL) at MOI = 10, followed by centrifugation at 1800 × *g*, 4 °C for 1 h.

### Flow cytometry

The expression of cell surface and intracytoplasmic markers was detected using flow cytometer. For extracellular staining, anti-human NKG2D-APC (Biolegend, 320808), anti-human CD69-APC (Biolegend, 310910), anti-human CD107a-PE (Biolegend, 328608), anti-human Tim-3-PE-cy7 (Biolegend, 345013), anti-human PD-1-PE (Biolegend, 329905), anti-human CD3-BV421 (Bioscience, 562877), anti-human CD45RA-FITC (Biolegend, 304106), anti-human CCR7-PE-cy7 (Biolegend, 353226), and Annexin V (Bioscience, 550474) were used for extracellular staining. For intracellular staining, CAR T cells were fixed and permeabilized using BD Cytofix/Cytoperm kit according to the recommendation of the manufacturer, and anti-human IFN-γ-Percp-cy5.5 (Invitrogen, 45-7319-42), anti-human Granzyme B-PE (Invitrogen, 12-8899-41) and anti-human p-ERK-APC (Invitrogen, 17-9109-41) were used for intracellular staining. Stained samples were acquired on a BD FACSAria and analyzed with FlowJo software.

### Quantitative real-time PCR

The total RNAs were collected from CAR T cells using TRIzol reagent according to the manufacturer’s instructions. Quantitative real-time PCR reactions were performed in an Applied Biosystems 7500 Real-Time PCR System. Reaction cycle conditions were as follows: 95 °C for 10 min of predenaturation conditions, 40 cycles at 95 °C for 15 s, 60 °C for 20 s, and 72 °C for 30 s. The primer sequences of the 4.1R gene were as follows: forward primer, 5′-TGGCTGGATTCCGCCAAAG-3′; reverse primer, 5′-CCTGCAACTATGTCCTGCCG-3′. All experiments were performed in triplicate.

### Western blot

Lysis buffer was used to extract the total protein, followed by centrifugation at 12,000 rpm, 4 °C for 10 min, and the supernatants were collected. The same amount of protein was subjected to 12.5% sodium-dodecyl sulfate polyacrylamide gel electrophoresis (SDS-PAGE) and transferred to polyvinylidene difluoridefilter (PVDF) membranes (Millipore, Bedford, MA, United States). The membranes were blocked with 5% dried skim milk in Tris-buffered saline/Tween 20 (TBST) for 1 h at room temperature, followed by probing with the primary antibodies against 4.1R and β-actin overnight at 4 °C. Subsequently, the membranes were washed three times with TBST on the shaker, and then incubated with the secondary antibody for 1 h at room temperature.

### Cytotoxicity assay

Flow cytometry was applied to evaluate the anti-tumor activity of CAR T cells. Firstly, SW1990, CAPAN2 and PANC28 cells (target cells) were stained with carboxyfluorescein succinimidyl ester (CFSE) and then seeded into low adsorption 96-plates at a density of 2 × 10^4^ cells/well. Secondly, non-transduced T cells or CAR T cells (effector cells) were added to each well to ensure an effector to target cell (*E*:*T*) ratio of 1:1, 3:1, or 9:1. After 16 h, the cell mixtures were collected, stained with anti-Annexin V antibodies and quantified by flow cytometry.

### Xenograft mouse model

Eight-week-old female NSG (NOD-PrkdcscidIL2rgtm1/Bcgen, Beijing Biocytogen Co., Ltd) mice were used in this current study. 6 × 10^6^ PANC28/luc cells were inoculated subcutaneously (s.c.) in the right flank of all the mice. For in vivo cytotoxicity experiments, 1 × 10^7^ CAR T cells were injected via the tail vein (intravenous; i.v.) into tumor-bearing mice. The bioluminescent imaging (BLI) was monitored using Xenogen-IVIS Imaging System (Caliper Life Sciences, Hopkinton, MA) once a week. All animal studies were carried out under protocols approved by the Institutional Animal Care and Use Committee of East China Normal University.

### Statistical analyses

All statistics were performed using Prism software (GraphPad) and expressed as mean ± SD. Data were compared using the one-way ANOVA test. Comparison of survival curves was done using the log-rank test. *P* values < 0.05 were considered significant. Each experiment consisted of at least three replicates per condition.

## Supplementary information

Supplementary figures
