# Supplementary material for: Suppression of 4.1R enhances the potency of NKG2D-CAR T cells against pancreatic carcinoma via activating ERK signaling pathway
Source: Oncogenesis. 2021 Sep 21;10(9):62. doi: 10.1038/s41389-021-00353-8 (PMC8455638; doi:10.1038/s41389-021-00353-8)
Supplement: Supplementary file 1 — Supplementary figures [file 41389_2021_353_MOESM1_ESM.doc]

**Supplemental Figures**

**Fig. S1:**

**Detection of cytotoxic activity in NKG2D-CAR T cells.** The expression of CD69 (**a**), release of IFN-γ (**b**) and Gzm B (**c**) as well as the expression of CD107a (**d**) were measured after co-incubating with CAPAN2 at a 9:1 ratio for 16h by flow cytometry (left). MFI and percentage were statistically analyzed and shown in column chart (middle and right) (n=3). Data were representative of three independent experiments. * P < 0.05, ** P < 0.01, NS, not significant.

Fig. S2.

**Detection of cytotoxic activity in NKG2D-CAR T cells.** The expression of CD69 (**a**), release of IFN-γ (**b**) and Gzm B (**c**) as well as the expression of CD107a (**d**) were measured after co-incubating with SW1990 at a 9:1 ratio for 16h by flow cytometry (left). MFI and percentage were statistically analyzed and shown in column chart (middle and right) (n=3). Data were representative of three independent experiments. NS, not significant.

Figure S3.

**The expression of P-ERK in NKG2D-CAR T cells after adding U0126.** Flow cytometry was used to detect the expression of P-ERK in absence and presence of 10 μM U0126 (**a**), and MFI was calculated (**b**) (n=3). Data were representative of three independent experiments. ** P < 0.01.
